# Dual inhibition of carbonic anhydrases VA and VII by silychristin and isosilybin A from *Silybum marianum*: A potential antiobesity strategy

**DOI:** 10.1002/ardp.202400966

**Published:** 2025-03-24

**Authors:** Emanuele Liborio Citriniti, Roberta Rocca, Giosuè Costa, Gioele Renzi, Fabrizio Carta, Claudiu T. Supuran, Stefano Alcaro, Francesco Ortuso

**Affiliations:** ^1^ Dipartimento di Scienze della Salute Università “Magna Græcia” di Catanzaro Catanzaro Italy; ^2^ Net4Science S.r.l. Università “Magna Græcia” di Catanzaro Catanzaro Italy; ^3^ Associazione CRISEA—Centro di Ricerca e Servizi Avanzati per l'Innovazione Rurale Catanzaro Italy; ^4^ NEUROFARBA Department, Sezione di Scienze Farmaceutiche University of Florence Florence Italy

**Keywords:** docking, dual inhibition, *h*CAs, milk thistle, obesity

## Abstract

Obesity is a global health crisis linked to chronic diseases like cardiovascular disease and type 2 diabetes. Its prevalence, even in low‐income countries, highlights the failure of traditional interventions. Safer pharmacological treatments are urgently needed, as many existing antiobesity drugs have been withdrawn due to severe side effects, leaving a critical therapeutic gap. A promising target in this context is human carbonic anhydrase V (*h*CA V), a mitochondrial enzyme that plays a key role in glucose homeostasis. Inhibiting *h*CA V has been shown to reduce lipogenesis and improve metabolic conditions. Natural plant extracts, such as silymarin from milk thistle, have demonstrated potential in managing obesity‐related metabolic syndromes by lowering triglycerides, reducing cholesterol levels, and improving liver function. Our computational studies have identified active compounds in silymarin that effectively inhibit *h*CA V, shedding light on a potential mechanism for its antiobesity effects. Building on these findings, our research further reveals that these compounds also inhibit carbonic anhydrase VII (*h*CA VII), enhancing their therapeutic potential. This dual inhibitory action addresses both metabolic dysregulation and oxidative stress. Notably, the antioxidant properties of *h*CA VII provide additional protection against obesity‐related complications by mitigating oxidative stress, a key contributor to the development of metabolic syndrome.

## Graphical abstract

Safer treatments for obesity are urgently needed, as many drugs were withdrawn due to their severe side effects. Targeting human carbonic anhydrase V (*h*CA V), the key to glucose homeostasis, shows promise. Silymarin from milk thistle is highlighted as a dual inhibitor of *h*CA V and VII, addressing metabolic dysregulation and oxidative stress, and offering new hope for obesity management.

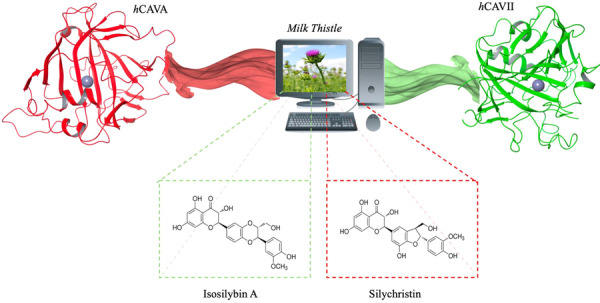

## 1 INTRODUCTION

Obesity is a pressing global public health concern that impacts people worldwide. It significantly contributes to chronic illnesses, including cardiovascular disease,^[^ ^1^ ^]^ certain types of cancer,^[^ ^2^ ^]^ nonalcoholic fatty liver disease,^[^ ^3^ ^]^ and type 2 diabetes mellitus.^[^ ^4^ ^]^ The global issue of obesity has reached epidemic proportions, with its prevalence tripling in several European nations and affecting a significant portion of the populace. Once considered problems primarily affecting affluent nations, obesity and its associated health risks are now spreading rapidly to lower income countries, posing an alarming global threat.^[^ ^5^, ^6^ ^]^ Obesity is a severe health condition that is challenging to treat with lifestyle and behavioral changes alone. While these adjustments can help alleviate some symptoms, they are often not sufficient for long‐term effectiveness.^[^ ^7^ ^]^ Adding pharmacological and surgical interventions to the treatment plan can lead to more significant results. However, many antiobesity medications have been developed and approved in the past, but they were later withdrawn due to severe side effects experienced by a significant number of patients. This lack of safe and effective long‐term options has created a growing interest in discovering new antiobesity drugs.^[^ ^5^ ^]^

Scientists are actively investigating potential targets for the development of antiobesity medications. One of the most promising targets identified is carbonic anhydrase V (*h*CA V), a mitochondrial enzyme directly involved in glucose homeostasis. With its favorable side effect profile, *h*CA V stands out as an attractive candidate for the development of new antiobesity therapies.^[^ ^8^, ^9^ ^]^ The human AC VA/B isoforms (*h*CA VA and VB) are important from a biological perspective because the mitochondrial membrane is impermeable to bicarbonate ions and only permeable to CO_2_. Moreover, there are no carriers for bicarbonate ions belonging to the SLC4A family. This means that de novo synthesis of bicarbonate within this compartment is essential for making substrates available to pyruvate carboxylase in the process of gluconeogenesis and lipogenesis, and carbamoyl phosphate synthase I in the process of ureagenesis in the liver. Thus, inhibiting *h*CA VA and VB could lead to a significant decrease in citrate levels, promoting a reduction in lipogenic phenomena. These isoenzymes may also be involved in insulin secretion and neuromodulation. Therefore, there is increasing interest in AC V as a potential target for different compounds with pharmacological activity.^[^ ^10^, ^11^ ^]^

In the last decade, extensive research has been conducted on plant extracts and their phytochemical constituents to evaluate their potential effectiveness in combating obesity.^[^ ^11^, ^12^ ^]^ Ethnopharmacology is an indispensable field for the discovery of new drugs and systematic screening of both pharmacological and phytochemical aspects.^[^ ^13^, ^14^ ^]^ Indeed, plants found in the wild are known to contain valuable plant secondary metabolites like polyphenols and terpenoids that have potential use as nutraceuticals, particularly in functional foods. Research on edible wild plants, using both in vitro and in vivo models, has revealed their capacity to contain active compounds that offer health benefits. Among the various plants of the Mediterranean basin, *Silybum marianum (L.)*, commonly known as milk thistle (MT), is very interesting for its beneficial effects on metabolic syndrome. It is an annual/biennial weed and a trusted herbal remedy for liver and biliary tract ailments. *S. marianum* is also known for its numerous health benefits, including antioxidant, lipid‐lowering, antihypertensive, antidiabetic, antiatherosclerotic, antiobesity, and hepatoprotective effects, making it an effective treatment for metabolic syndrome.^[^ ^15^ ^]^

The extract of *S. marianum*, commonly known as Silymarin, is primarily used to treat liver‐related disorders. This substance exhibits a variety of reported activities, including antioxidant, antifibrotic, regenerative, choleretic, hepatoprotective, immunostimulatory, and anti‐inflammatory effects. Silymarin is a standardized dry extract derived from the seeds of the *S. marianum* plant, predominantly composed of flavonolignans, which make up about 70%–80% of its total weight.^[^ ^16^ ^]^ Additionally, a combination of flavonoids, including polymerized and oxidized polyphenolic compounds, can be observed. Flavonolignans are a relatively limited subgroup of compounds where the flavonoid part of the molecule is fused with a lignan. Silybin, isosilybin (A and B), silydianin, and silychristin are the primary flavonolignans found in silymarin, and they are the most biologically active compounds within the extract, despite the identification of unconventional flavolignans.^[^ ^17^ ^]^ Although numerous studies have detailed the analytical separation and quantification of silymarin components in extracts from various plant parts, seasons, and geographic locations, there is currently no comprehensive comparison of flavonolignan profiles in different silymarin preparations.^[^ ^18^, ^19^, ^20^ ^]^ Studies have shown that the extract of Silymarin can reduce triglyceride levels in patients with nonalcoholic fatty liver disease (NAFLD) who consumed daily doses of 7.1 g of MT for 3 months as a source of silymarin (210 mg per day).^[^ ^21^ ^]^ An in vivo study on obese mice with metabolic syndrome and consequent type 2 diabetes mellitus and cardiovascular pathologies such as hypertension showed that the intake of silymarin reduced total body weight without affecting lean body weight. It also reduced serum levels of total cholesterol, triglycerides, and low‐density lipoproteins (LDL) while improving insulin resistance in obese mice. Furthermore, the extract improved evident liver damage and hepatic steatosis in the group fed with a high‐fat diet (HFD). Silymarin enhanced the degree of hepatic steatosis and inflammation in liver tissue, reducing serum Alanine Aminotransferase and Aspartate Aminotransferase) levels. The results suggest that silymarin could effectively inhibit HFD‐induced hepatic steatosis and improve liver function. Therefore, the extract of Silymarin obtained from the MT plant could represent an effective aid in the prevention and treatment of metabolic syndromes related to obesity and related disorders.^[^ ^22^ ^]^

In light of the above, we have decided to use computational methods to explore the most biologically active compounds found in MT extract. We aim to evaluate the efficacy of these compounds as *h*CA VA inhibitors by examining how they interact with the three‐dimensional (3D) structure of carbonic anhydrase isoforms found in the Protein Data Bank (PDB). To achieve this, we used a structure‐based approach that involved docking and molecular dynamics (MDs) simulations. After identifying the top compounds from the in silico analysis, we conducted enzymatic assays to validate the results. This study revealed the molecular mechanism behind the antiobesity effect of two active compounds from *S. marianum (L.)* by demonstrating their interaction and inhibition of *h*CA VA.^[^ ^15^ ^]^

## 2 RESULTS AND DISCUSSION

### 2.1 Molecular docking

In this study, we conducted docking simulations to evaluate the interactions of the six compounds contained in the *S. marianum* extract with the homology modeling structure of *h*CA VA. A threshold G‐score of –4.48 kcal/mol, derived from the best docking pose of acetazolamide (AAZ) as a positive control on *h*CA VA, was established. Out of the six compounds, only two ligands exhibited G‐scores lower than AAZ, suggesting their potential inhibitory activity against the target enzyme (Table 1). Specifically, silychristin and isosylibin A were identified as potential inhibitors of *h*CA VA, with G‐scores of –4.63 and –4.53 kcal/mol, respectively. The complexes of silychristin and isosilybin A with *h*CA VA were further analyzed thermodynamically using the molecular mechanics/generalized Born surface area (MMGBSA) method.^[^ ^23^ ^]^ To assess their selectivity for the mitochondrial isoform, we also analyzed their complexes with other *h*CA isoforms for which crystal structures are available in the PDB. Specifically, we compared the binding free energy values (ΔG_bind_) of the two compounds from the *S. marianum* extract to those of the reference ligand AAZ, used as a positive control (Table 2).

**Table 1:** Pubchem ID (CID), common name, 2D structure, and G‐Scores (kcal/mol) value for the six most abundant molecules in milk thistle extracts studied as potential *h*CA VA inhibitors.

Pubchem CID	Common name	2D Structure	G‐Score (kcal/mol)
441764	Silychristin	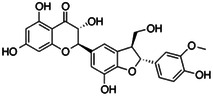	–4.63
11059920	Isosylibin A	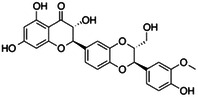	–4.53
31553	Silybin A	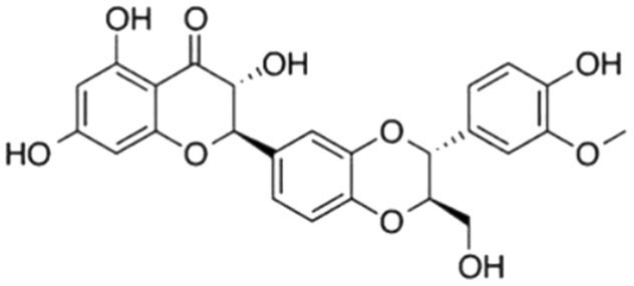	–4.44
11982272	Silidianin	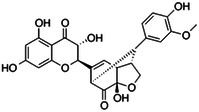	–4.43
10885340	Isosylibin B	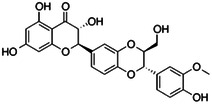	–4.08
1548994	Sylibin B	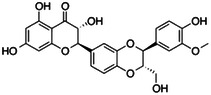	–3.78

*Note*: Only silychristin and isosylibin A exceeded the cut‐off value of the AAZ.

Abbreviation: AAZ, acetazolamide.

**Table 2:** Δ*G*_bind_ values of silychristin and isosylibin A complexed with each *h*CA isoform.

*h*CA isoforms	Silychristin Δ*G*_bind_ (kcal/mol)	Isosylibin A Δ*G*_bind_ (kcal/mol)
*h*CA I	–41.00	–36.25
*h*CA II	–33.21	–33.16
*h*CA VA	–27.02	–28.53
*h*CA VII	–48.35	–41.77
*h*CA IX	–22.08	–21.37
*h*CA XII	–36.48	–39.04

*Note*: All ΔG values are reported in kcal/mol.

Abbreviation: hCA, human carbonic anhydrase.

The resulting data proved highly valuable in elucidating the key forces driving the interactions between the selected compounds and the *h*CA isoforms, providing insights into their underlying selectivity. Notably, our compounds exhibited better binding free energy (Δ*G*_bind_) values compared with AAZ for both the *h*CA VA (the primary isoform of interest) and VII, an isoform involved in oxidative stress protection and neuropathic pain (Table 2).^[^ ^24^, ^25^ ^]^ Specifically, silychristin and isosilybin A demonstrated Δ*G*_bind_ values of –27.02 and –28.53 kcal/mol, respectively, for *h*CA VA, and –48.35 and –41.77 kcal/mol for *h*CA VII. Conversely, these compounds showed lower affinity toward all other isoforms compared with AAZ, underscoring their potential selectivity for *h*CA VA and *h*CA VII.

Regarding the components related to Δ*G*_bind_, in both *h*CAs isoforms complexes, we identified the lipophilic (Δ*G*_lipo_) and van der Waals (Δ*G*_vdW_) contributions as the most significant for the two compounds found in MT, compared with AAZ. The Δ*G*_Lipo_ values for silychristin and isosilybin A are very similar, at –14.82 and –14.79 kcal/mol, respectively, while the Δ*G*_vdW_ values are –38.08 and –39.72 kcal/mol, respectively. In comparison, the binding of AAZ to both *h*CA VA and *h*CA VII active sites is mainly influenced by electrostatic interactions, as evidenced by its more favorable Δ*G*_Coul_ values of –50.84 and –77.97 kcal/mol. Among the *hits*, isosilybin A exhibited a significant electrostatic contribution with a Δ*G*_Coul_ value of –32.32 kcal/mol when complexed with *h*CA VA. On the other hand, silychristin demonstrated an even greater electrostatic contribution with a Δ*G*_Coul_ value of –52.50 kcal/mol when complexed with *h*CA VII. By analyzing the solvation penalty, we observed the highest Δ*G*_SolvGB_ values for isosilybin A (49.93 kcal/mol) when complexed with *h*CA VA, and for AAZ (64.83 kcal/mol) when complexed with *h*CA VII.

**Figure 1:**
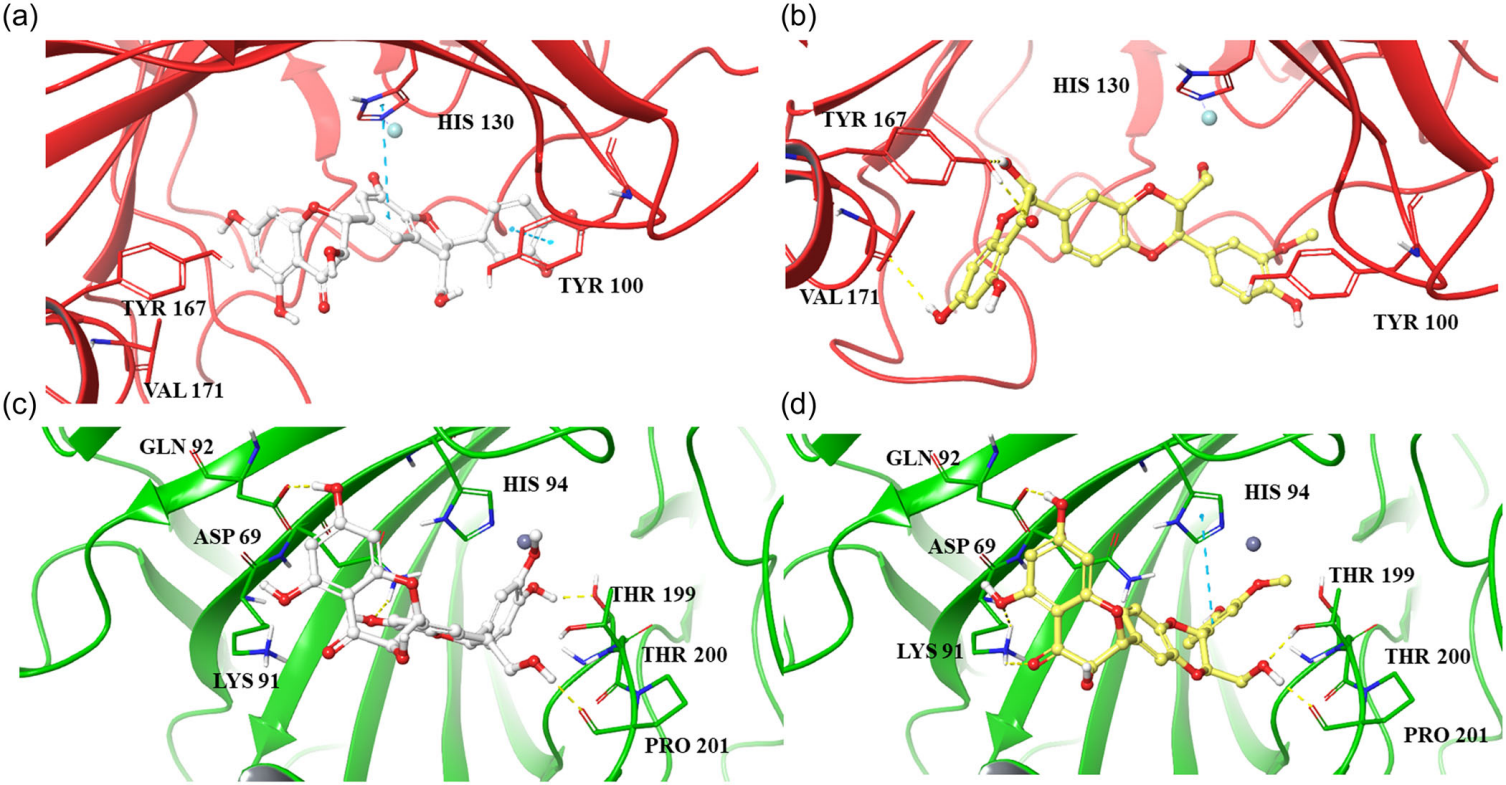
Three‐dimensional (3D) representation of human carbonic anhydrase (*h*CA) VA and *h*CA VII complexed with (a, c) silychristin and (b, d) isosilybin A, respectively. Silychristin and isosilybin A are depicted as gray and yellow ball‐and‐stick models, while the zinc ion is shown as a gray sphere. *h*CA VA and *h*CA VII are represented as red and green cartoons, respectively. Additionally, the amino acid residues involved in the most significant interactions with the ligands are shown as red and green sticks for *h*CA VA and *h*CA VII, respectively. H‐bonds, salt bridges, and stacking interactions are illustrated as yellow, red, and cyan dashed lines, respectively.

By evaluating the docking poses, we observed distinct binding modes for both silychristin and isosilybin A in *h*CA VA and *h*CA VII (Figure 1 and Supporting Information S2: Figure S1). Specifically, in the best docking pose with the *h*CA VA, silychristin formed two π–π interactions: one between its hydroxyl‐methoxyphenyl moiety and TYR 100, and another between its dihydrobenzofuran ring and HIS 130. Furthermore, the dihydrobenzofuran ring also participated in electrostatic interactions with the zinc ion (Figure 1a and Supporting Information S2: Figure S1A). Conversely, the docking analysis for the *h*CA VII active site revealed the formation of four hydrogen bonds (H‐bonds) involving the hydroxyl groups of the silychristin and the residues ASP 69, GLN 92, THR 199, and PRO 201. Additionally, its hydroxyl‐methoxyphenyl moiety was oriented toward the zinc ion, facilitating potential electrostatic interactions (Figure 1b, Supporting Information S2: Figure S1B). For isosilybin A, the best docking pose with *h*CA VA revealed that the ligand was well‐accommodated within the catalytic site, primarily stabilized by three hydrogen bonds (H‐bonds) between the 2,3‐dihydrochromen‐4‐one group and the residues TYR167 and VAL171. Furthermore, the 1,4‐benzodioxine moiety formed favorable electrostatic interactions with the zinc ion (Figure 1c, Supporting Information S2: Figure S1C). On the other hand, in the *h*CA VII complex, docking analysis revealed a prominent cation–π interaction between the hydroxymethoxyphenyl group of isosilybin A and HIS94, along with two H‐bonds with THR200 and PRO201, and two additional H‐bonds with ASP69 and LYS91 through the dihydrocoumarin moiety (Figure 1d and Supporting Information S2: Figure S1D).

### 2.2 MDs

MDs were performed to investigate the Induced‐Fit (IF) process triggered by the binding of silychristin and isosilybin A to *h*CA VA and *h*CA VII, starting from their best docking poses. Compared with AAZ, the ligands extracted from *S. marianum* displayed similar behavior on both CA isoforms. This is evidenced by the comparable root mean square deviation (RMSD) values calculated for the protein‐heavy atoms (Supporting Information S2: Figure S2A‐B).

Regarding the RMSD trend calculated for the ligand‐heavy atoms, both compounds demonstrated a rearrangement in their binding mode compared with AAZ (Supporting Information S2: Figure S2C‐D). This observation suggests a more pronounced IF process for the ligands than for the protein. Specifically, isosilybin A showed an initial adjustment in both isoform complexes that persisted throughout the MDs (Supporting Information S2: Figure S2C‐D, green line). Conversely, silychristin exhibited behavior similar to AAZ during the first 130 ns of MD simulations for the *h*CA VA complex (Supporting Information S2: Figure S2C, blue line), showing only minor changes toward the end. However, in the *h*CA VII complex, silychristin underwent significant alterations in its binding mode after 100 ns, reaching RMSD values of 6 Å (Supporting Information S2: Figure S2D, blue line). The higher RMSD values and greater fluctuations for isosilybin A and silychristine in both figures suggest a more dynamic interaction with the protein, undergoing significant conformational changes during the binding process. In contrast, AAZ's stable RMSD values indicate a more rigid and consistent binding mode, reflecting less structural adaptation in response to the protein environment. These data underscore the importance of considering the flexibility and IF potential of ligands in drug design and binding studies.

**Figure 2:**
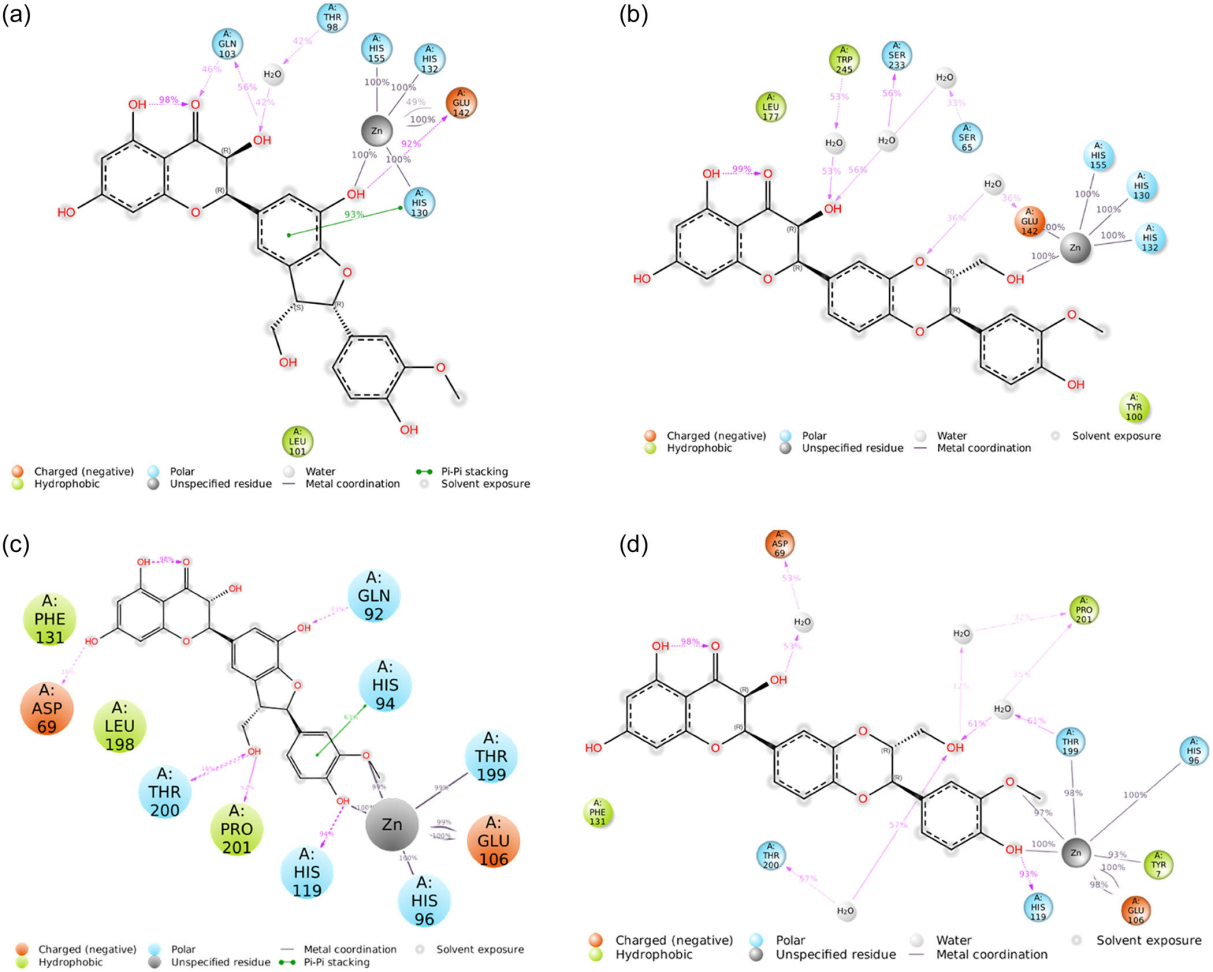
Ligand atom interactions with the human carbonic anhydrase (*h*CA) VA and *h*CA VII residues, for silychristin (a–c) and isosilybin A (b–d), respectively. Only interactions that occur more than 30.0% of the simulation time in 200 ns of the trajectory are shown.

Finally, we conducted a detailed analysis of the interactions between the ligand atoms and the protein residues throughout the simulation (Figure 2). Specifically, our focus was on interactions that occurred for more than 30.0% of the total simulation time.

In our MD simulations involving *h*CA VA, we observed two distinct interaction patterns for our compounds, with the exception of the consistent electrostatic interaction between a hydroxyl group of both compounds and the zinc ion throughout the entire simulation (Figure 2a,b). Two notable interactions between silychristin and *h*CA VA occurred during 92% of the simulation time. These included a π–π interaction between the 2,3‐dihydrobenzofuran moiety and HIS 130, and an H‐bond formed between the phenolic group of the same moiety and GLU 142. Moreover, the hydroxychroman‐4‐one moiety engaged in two H‐bonds with GLN 103, observed for 56% and 46% of the simulation, respectively, and formed a water bridge with THR 98 (Figure 2a). On the other hand, the binding mode of isosilybin A was defined by several water bridges with the residues SER 65, GLU 142, SER 233, and TRP 245, as well as hydrophobic interactions with TYR 100 and LEU 177 (Figure 2b). Despite the high RMSD trend for both compounds in *h*CA VII complex (Supporting Information S2: Figure S2D), silychristin and isosilybin A have strong and stable interactions within this binding pocket (Figure 2c,d). The ligands establish electrostatic interactions with the Zn^2+^ and form multiple H‐bonds with high occupancy, indicating their significance in maintaining the binding conformation after the IF process. In particular, the MD simulations of the *h*CA VII complexes revealed persistent electrostatic interactions between the o‐methoxy‐phenolic portion of both compounds and the Zn²⁺ ion, maintained consistently throughout the entire simulation (Figure 2c,d). Specifically, the isosilybin A binding mode was characterized by different water bridges involving the residues ASP 69, THR 199, THR 200, and PRO 201, as well as an important H‐bond with HIS 119 (Figure 2d). Similarly, silychristin maintained an H‐bond with HIS 119 for almost 90% of the MD simulations. Additionally, its binding mode exhibited four other stable H‐bonds with the residues GLN 92, ASP 69, THR 200, and PRO 201, along with a π–π interaction with HIS 94 (Figure 2c).

### 2.3 Carbonic anhydrase inhibition assays

The inhibition profiles of silychristin and isosylibin A on the physiologically relevant *h*CAs I, II, VA, VII, IX, and XII were determined through the stopped‐flow CO_2_ hydrase assay^[^ ^26^ ^]^ and were compared with the commercially available AAZ as reference. Inhibition constants (*K*_I_) were reported in Table 3.

**Table 3:** Inhibition data of silychristin and isosylibin A and the references AAZ on hCAs I, II, VA, VII, IX, and XII.^[^ ^26^ ^]^

*K*_I_ (µM)^a^						
Compounds	*h*CA I	*h*CA II	*h*CA VA	*h*CA VII	*h*CA IX	*h*CA XII
Silychristin	>100	>100	5.25	0.90	>100	59.8
Isosylibin A	>100	>100	0.92	0.94	>100	76.6
AAZ	0.25	0.012	0.063	0.003	0.026	0.006

Abbreviation: AAZ, acetazolamide.

^a^ Mean from 3 different assays by a stopped‐flow technique. Errors were in the range of ± 5–10% of the reported values.

As shown in Table 3, silychristin and isosylibin A were ineffective inhibitors on the broadly expressed *h*CAs I, II, and on the tumor‐associated IX isoforms (*K*_I_ *s* > 100 μM). Conversely, close matching values were obtained for the central nervous system (CNS) related *h*CA VII as silychristin and isosylibin A have *K*_I_ values of 0.90 and 0.94 μM, respectively. Substantial kinetic discrimination was observed for the mitochondrial *h*CA VA, being the Isosylibin A 5.7‐fold more effective inhibitor when compared with silychristin (i.e., *K*_I_ *s* of 5.25 and 0.92 μM for silychristin and isosylibin A, respectively). Medium micromolar values were obtained for both compounds on the *h*CA XII isoform (i.e.,*K*_I_ *s* of 59.8 and 76.6 μM for silychristin and isosylibin A, respectively).

In agreement with the predicted binding modes of silychristin and isosylibin A for the hCAs, the experimental in vitro inhibition potencies were far higher than the reference drug AAZ which possesses the prototypic CA inhibitory moiety of the primary sulfonamide type. For instance, the *h*CA VA submicromolar inhibitor isosylibin A was 14.6‐fold less effective when compared with AAZ (i.e., *K*_I_ of 0.063 µM), and such difference was up to 83.3‐fold higher for silychristin when tested on the same enzyme. Data in Table 3 clearly showed that the high effectiveness of both natural products toward the *h*CA VII was not comparable to the reference AAZ, which exhibited a *K*_I_ of 3.0 nM. Even more drastic differences were reported for the tumor‐associated *h*CA XII isoform as silychristin and Isosylibin A were 10.000‐ and 12.777‐fold less effective when compared with AAZ (i.e. *K*_I_s of 59.8, 76.6, and 0.006 µM, respectively).

It is, therefore, clear that silychristin is highly effective and selective for the *h*CA VII being the associated *K*_I_ value of 0.90 μM. The same potency was reported for isosylibin A, which is an equipotent inhibitor of both the *h*CAs VA and VII (*K*_I_ *s* of 0.92 and 0.94 μM for *h*CA VA and VII, respectively).

## 3 CONCLUSION

The results of this study highlight the potential of two compounds from *S. marianum* (MT)—silychristin and isosilybin A—as selective inhibitors of specific *h*CA isoforms, particularly *h*CA VA and *h*CA VII. This conclusion is supported by an integrative approach combining docking simulations, MD simulations, and in vitro inhibition assays. The docking analysis suggests a potential affinity of these compounds for *h*CA VA, revealing a novel mechanism of action and positioning them as promising candidates for antiobesity pharmacology. The biophysical assays confirmed their inhibitory activity against *h*CA VA, reinforcing their potential as antiobesity agents. Computational studies further predicted the selectivity of silychristin and isosilybin A for *h*CA VA and VII, which was validated by the biophysical assays. Thermodynamic and interaction analyses showed that the inhibitory activity of these compounds is driven by a combination of lipophilic, Van der Waals, and electrostatic interactions, along with extensive hydrogen bonding. The lipophilic and Van der Waals forces were the primary contributors, providing significant stabilization in the binding sites of both *h*CA VA and VII. While AAZ exhibited stronger overall electrostatic interactions, silychristin, and isosilybin A formed extensive hydrogen bonding networks, particularly with residues around the zinc ion, as well as several water bridges, contributing to their stable and selective binding profiles.

Regarding the electrostatic interactions with the zinc ion observed in the best docking poses, it is important to note that the docking results may not fully match experimental literature data, partly due to the challenges of accurately modeling water molecules in the active site.^[^ ^27^, ^28^ ^]^ Water molecules play a critical role in stabilizing ligand interactions, but their dynamic behavior is often oversimplified or overlooked in docking simulations.^[^ ^29^ ^]^ Additionally, because docking relies on molecular mechanics, it may not fully capture electronic effects such as polarization and charge transfer, which are better addressed through quantum mechanical methods.^[^ ^30^ ^]^ These limitations can lead to some variability in the predicted binding affinities and interaction profiles. Despite these limitations, docking remains a valuable and widely used tool for predicting ligand–enzyme interactions, providing rapid insights into binding poses, interaction patterns, and relative binding affinities. In this study, the docking results offer a strong foundation for understanding the key forces behind the inhibitory activity of the compounds and highlight their potential selectivity, which is further corroborated by complementary analyses like MDs simulations.^[^ ^31^, ^32^ ^]^

The selective inhibition of *h*CA VA and VII by silychristin and isosilybin A offers promising therapeutic opportunities, particularly for conditions associated with these isoforms. *h*CA VA plays a critical role in mitochondrial function, and its inhibition could have significant therapeutic potential for disorders linked to mitochondrial dysfunction, including neurodegenerative diseases, metabolic disorders, and age‐related conditions. Targeting *h*CA VA, silychristin, and isosilybin A may provide a novel approach to mitigate symptoms or slow disease progression by enhancing mitochondrial efficiency and optimizing cellular energy production.

Similarly, *h*CA VII, predominantly expressed in the central nervous system (CNS), is involved in regulating oxidative stress, pain pathways, and neuroinflammation.^[^ ^24^, ^33^ ^]^ Inhibition of *h*CA VII could be highly beneficial for conditions involving oxidative damage, neuropathic pain, and neurodegenerative diseases such as Alzheimer's, Parkinson's, and multiple sclerosis. The ability of silychristin and isosilybin A to selectively target *h*CA VII suggests that these compounds may offer a more targeted therapeutic strategy, potentially reducing the harmful effects of oxidative stress and neuroinflammation in the brain and spinal cord.^[^ ^24^ ^]^

A key advantage of these compounds is their high selectivity for *h*CA VA and VII, which minimizes the risk of off‐target effects commonly seen with nonselective carbonic anhydrase inhibitors. *h*CAs I, II, and IX are involved in a range of physiological processes, and nonselective inhibition of these isoforms can lead to adverse side effects, such as electrolyte imbalances, gastrointestinal issues, or cognitive disturbances. The lack of significant activity against these isoforms in silychristin and isosilybin A significantly lowers the risk of such complications, positioning these compounds as safer alternatives for therapeutic use. This selective inhibition not only enhances the therapeutic potential of these compounds but also underscores their specificity as promising drug candidates, with a reduced risk of unwanted side effects—an essential feature in the development of drugs for chronic and complex diseases.

This study highlights the promising potential of silychristin and isosilybin A as selective inhibitors of *h*CA VA and *h*CA VII, offering therapeutic promise for conditions linked to mitochondrial dysfunction and oxidative stress. The integrative approach, combining docking simulations, MD, and biophysical assays, has demonstrated the compounds’ high selectivity and stable binding profiles, supporting their suitability for drug development. Finally, the lack of significant off‐target effects, particularly on *h*CA isoforms I, II, and IX, strengthens the case for their use in treating chronic and complex diseases, making them strong candidates for further clinical investigation.

## 4 EXPERIMENTAL

### 4.1 Receptor preparation

Due to the unavailability of experimental structures of human carbonic anhydrase V (*h*CA V), we employed the homology model previously developed and validated in our study to advance our investigation into this isoform and its inhibitors.^[^ ^24^ ^]^ Additionally, we examined the interaction of the selected compounds with other isoforms to evaluate their selectivity for our target isoform. Thus, we obtained the crystallographic structure of various isoforms of hCAs from the Protein Data Bank (PDB).^[^ ^34^ ^]^ After careful consideration, we selected the models with the following PDB code for our analysis: 7QOD for *h*CA I,^[^ ^35^ ^]^ 6SBL for *h*CA II,^[^ ^36^ ^]^ 6SDT for *h*CA VII,^[^ ^37^ ^]^ 5FL4 for *h*CA IX,^[^ ^38^ ^]^ and 5MSA for *h*CA XII.^[^ ^39^ ^]^ The Protein Preparation Wizard tool^[^ ^40^ ^]^ to prepare the three‐dimensional (3D) structures of all the *h*CA isoforms, by applying OPLS3 as a force field.^[^ ^41^ ^]^ All models were refined for increased accuracy using the advanced Prime module to reconstruct missing side chains.^[^ ^42^ ^]^ Hydrogen atoms were added, and side‐chain protonation states were assigned at pH 7.4. Any crystallographic buffer components and water molecules remaining in the model were removed. Subsequently, the co‐crystallized ligands were set as centroids for generating the rigid receptor grid, which was defined by a 10 × 10 × 10 Å inner box.

### 4.2 Ligands preparation

From Pubchem,^[^ ^43^ ^]^ we downloaded the six CID (Chemical Identifier) of the most prevalent and biologically active compounds extracted from the *S. marianum* species. These six molecules were prepared using Ligprep at pH 7.4, maintaining default chirality.^[^ ^44^ ^]^ The molecules were minimized and optimized using the OPLS3 as a force field for subsequent docking studies. Acetazolamide (AAZ) was prepared using the same protocol and served as a positive control in this process.

### 4.3 Docking simulations

For our docking simulations, we used Glide *ver*. 7.8 software applying the Standard‐Precision (SP) protocol.^[^ ^45^ ^]^ We generated 10 poses for each ligand using default settings and selected the best binding mode based on the docking scoring function (G‐Score). Thus, the re‐docking analysis was performed for each PDB model to verify the reliability of our docking protocol for this model (Supporting Information S2: Table S1). First, we filtered the compounds with promising affinity for *h*CA V based on their G‐score values. Specifically, we used the best G‐score (–4.48 kcal/mol) obtained from the docking of AAZ with the *h*CA VA isoform as the cut‐off (Supporting Information S2: Table S2). Compounds that successfully cleared the *h*CA V filter underwent evaluation on the remaining isoforms, with AAZ serving as the reference once more. Specifically, a thermodynamic analysis was performed on the best binding pose of the compounds across all isoforms. In fact, the MMGBSA method has been widely used in the study of protein–ligand interactions.^[^ ^46^, ^47^, ^48^, ^49^ ^]^ Its application allowed the calculation of binding free energies (ΔG_bind_) of the complexes of the selected compounds, providing valuable insight into their thermodynamic stability.

### 4.4 MDs simulations

The best complexes of *h*CA VA and *h*CA VII with the two most promising compounds from *S. marianum* (silychristin and isosylibin A), as selected from docking, were submitted to 200 ns of MDs using Desmond *ver*. 4.4.^[^ ^50^ ^]^ All systems were enclosed within an orthorhombic box with a 10 Å thickness, containing the water solvent. Specifically, we employed the TIP3P water model parameters.^[^ ^51^ ^]^ The charge of all systems was neutralized by adding Na^+^ as counterions. We optimized the solvated model for accuracy and then relaxed it using the Martyna–Tobias–Klein isobaric‐isothermal ensemble (MTK_NPT). Equilibration was ensured through both the NVT ensemble at 10 K and the NPT ensemble at 300 K and 1 atm with the Berendsen thermostat–barostat. Our analysis involved sampling trajectory frames every 200 ps and utilizing tools such as the Simulation Interaction Diagram and the Simulation Event Analysis. These tools facilitated a thorough exploration of the complex geometric and thermodynamic properties underlying the trajectories obtained. Additionally, we employed the same rigorous methodology to investigate both *h*CA isoforms complexes, alongside the well‐established inhibitor AAZ, which had been utilized in the prior docking process. This comprehensive approach ensured the reliability and consistency of the findings across all systems studied.

### 4.5 Carbonic anhydrase inhibition assays

An applied photophysics stopped‐flow instrument was used to evaluate the ability of the test compounds to inhibit the CA‐catalyzed CO_2_ hydration.^[^ ^26^ ^]^ Phenol red (at a concentration of 0.2 mM) was used as an indicator, working at the absorbance maximum of 557 nm, with 20 mM 4‐(2‐hydroxyethyl)‐1‐piperazineethanesulfonic acid (pH 7.4) as a buffer, and 20 mM Na_2_SO_4_ (to maintain constant ionic strength), following the initial rates of the CA‐catalyzed CO_2_ hydration reaction for a period of 10–100 s. The CO_2_ concentrations ranged from 1.7 to 17 mM for the assessment of the kinetic parameters and inhibition constants. Enzyme concentrations varied between 5 and 12 nM.^[^ ^52^, ^53^ ^]^ For each inhibitor, at least six traces of the initial 5%–10% of the reaction were used to determine the initial velocity. The uncatalyzed rates were calculated in the same manner and subtracted from the total observed rates. Stock solutions of each inhibitor (0.1 mM) were prepared in distilled–deionized H_2_O, and dilutions up to 0.01 nM were done thereafter with the assay buffer. Inhibitor and enzyme solutions were preincubated together for 15 min at room temperature before the assay, to allow for the formation of the E–I complex.^[^ ^53^ ^]^ The inhibition constants were obtained by nonlinear least‐squares methods using PRISM 3^[^ ^26^ ^]^ and the Cheng–Prusoff equation as reported earlier^[^ ^54^, ^55^ ^]^ and represent the mean from at least three different determinations. *h*CAs I and II were purchased from Merck, while *h*CAs IX and XII are recombinant and obtained in‐house.^[^ ^56^, ^57^, ^58^, ^59^, ^60^ ^]^

## CONFLICTS OF INTEREST STATEMENT

The authors declare no conflicts of interest.

## Supporting information

Supporting information.

Supporting information.

## Data Availability

Data that support the findings of this study are available in the supplementary material of this article.
