# Supplementary material for: Dual inhibition of carbonic anhydrases VA and VII by silychristin and isosilybin A from Silybum marianum: A potential antiobesity strategy
Source: Arch Pharm (Weinheim). 2025 Mar 24;358(3):e2400966. doi: 10.1002/ardp.202400966 (PMC11931350; doi:10.1002/ardp.202400966)
Supplement: Supplementary file 1 — Supporting information. [file ARDP-358-e2400966-s001.doc]

**Supplemental Material: Novel Compounds and Biological Screening Results**

**Dual Inhibition of Carbonic Anhydrases VA and VII by Silychristin and Isosilybin A from Silybum marianum: A Potential Anti-Obesity Strategy**

Emanuele Liborio Citriniti^1^, Roberta Rocca,^1,2,3*^ Giosuè Costa^1,2^, Gioele Renzi^4^, Fabrizio Carta,^4^ Claudiu T. Supuran,^4^ Stefano Alcaro,^1,2,3^ Francesco Ortuso^1,2^

1 Dipartimento di Scienze della Salute, Università “Magna Græcia” di Catanzaro, Viale Europa, 88100 Catanzaro, Italy;

2 Net4Science S.r.l., Università “Magna Græcia” di Catanzaro, Viale Europa, 88100 Catanzaro, Italy;

3 Associazione CRISEA—Centro di Ricerca e Servizi Avanzati per l’Innovazione Rurale, Località Condoleo di Belcastro, 88055 Catanzaro, Italy;

4 NEUROFARBA Department, Sezione di Scienze Farmaceutiche, University of Florence, Via Ugo Schiff 6, 50019 Florence, Italy;

*Correspondence:

PhD, Roberta Rocca, Dipartimento di Scienze della Salute, Università degli Studi “Magna Græcia” di Catanzaro, Campus “Sal-vatore Venuta”, Viale Europa, 88100, Catanzaro, Italy

Email: [rocca@unicz.it](mailto:rocca@unicz.it);

|  |  | **KI (µM)*** | | | | | |
| --- | --- | --- | --- | --- | --- | --- | --- |
| ***Hits*** | **InChI codes** | ***h*CA I** | ***h*CA II** | ***h*CA VA** | ***h*CA VII** | ***h*CA IX** | ***h*CA XII** |
| **Sylichristin** | InChI=1S/C25H22O10/c1-33-18-6-10(2-3-15(18)28)23-14(9-26)13-4-11(5-17(30)25(13)35-23)24-22(32)21(31)20-16(29)7-12(27)8-19(20)34-24/h2-8,14,22-24,26-30,32H,9H2,1H3/t14-,22+,23+,24-/m1/s1 | >100 | >100 | 5.25 | 0.90 | >100 | 59.8 |
| **Isosylibin A** | InChI=1S/C25H22O10/c1-32-17-6-11(2-4-14(17)28)24-20(10-26)33-18-7-12(3-5-16(18)34-24)25-23(31)22(30)21-15(29)8-13(27)9-19(21)35-25/h2-9,20,23-29,31H,10H2,1H3/t20-,23+,24-,25-/m1/s1 | >100 | >100 | 0.92 | 0.94 | >100 | 76.6 |
