# Supplementary material for: Dual inhibition of carbonic anhydrases VA and VII by silychristin and isosilybin A from Silybum marianum: A potential antiobesity strategy
Source: Arch Pharm (Weinheim). 2025 Mar 24;358(3):e2400966. doi: 10.1002/ardp.202400966 (PMC11931350; doi:10.1002/ardp.202400966)
Supplement: Supplementary file 2 — Supporting information. [file ARDP-358-e2400966-s002.docx]

**Supporting Information**

**Dual Inhibition of Carbonic Anhydrases VA and VII by Silychristin and Isosilybin A from Silybum marianum: A Potential Anti-Obesity Strategy**

Emanuele Liborio Citriniti^1^, Roberta Rocca,^1,2,3*^ Giosuè Costa^1,2^, Gioele Renzi^4^, Fabrizio Carta,^4^ Claudiu T. Supuran,^4^ Stefano Alcaro,^1,2,3^ Francesco Ortuso^1,2^

1 Dipartimento di Scienze della Salute, Università “Magna Græcia” di Catanzaro, Viale Europa, 88100 Catanzaro, Italy;

2 Net4Science S.r.l., Università “Magna Græcia” di Catanzaro, Viale Europa, 88100 Catanzaro, Italy;

3 Associazione CRISEA—Centro di Ricerca e Servizi Avanzati per l’Innovazione Rurale, Località Condoleo di Belcastro, 88055 Catanzaro, Italy;

4 NEUROFARBA Department, Sezione di Scienze Farmaceutiche, University of Florence, Via Ugo Schiff 6, 50019 Florence, Italy;

*Correspondence:

PhD, Roberta Rocca, Dipartimento di Scienze della Salute, Università degli Studi “Magna Græcia” di Catanzaro, Campus “Sal-vatore Venuta”, Viale Europa, 88100, Catanzaro, Italy

Email: [rocca@unicz.it](mailto:rocca@unicz.it);

**Contents**

**Table S1.** Redocking analysis. PDB code, 2D structure of co-crystallized ligand and redocking RMSD value for each *h*CA isoform employed in the study. RMSD values are reported as Å..

**Table S2.** G-score and ΔG_bind_ values of Acetazolamide (AAZ) complexed to all *h*CA isoforms used in this study. G-score and ΔG_bind_ values are reported as Kcal/mol.

**Table S3.** Energetic components related to ΔG_bind_ (ΔG_Coul_, ΔG_lipo_, ΔG_solvGB_, ΔG_vdW_) for Acetazolamide (AAZ), silychristin and isosylibin A complexed with *h*CA VA and VII. All ΔG_bind_ values are reported as Kcal/mol.

**Figure S1.** 2D representation of *h*CA VA and *h*CA VII complexed with (A, C) silychristin and (B, D) isosilybin A, respectively. H-bonds, electrostatic, and stacking interactions are illustrated as magenta, red, and green lines, respectively.

**Figure S2**. **A-B)** Plot of the RMSD values calculated on the heavy atoms of *h*CA VA (A) and *h*CA VII (B) over 200 ns of MD simulations. The complexes studied include AAZ (red line), isosilybin A (green line), and silychristine (blue line). **C-D)** Plot of the RMSD values calculated on the heavy atoms for AAZ (red line), isosilybin A (green line), and silychristine (blue line) complexed with *h*CA VA (A) and *h*CA VII (B) over 200 ns of MD simulations.

**Table S1.** Redocking analysis. PDB code, 2D structure of co-crystallized ligand and redocking RMSD value for each *h*CA isoform employed in the study. RMSD values are reported as Å.

| ***h*CA isoform** | **PDB code** | **2D Structure** | **RMSD value**  ***(Å)*** |
| --- | --- | --- | --- |
| *h*CA I | 7QOD | 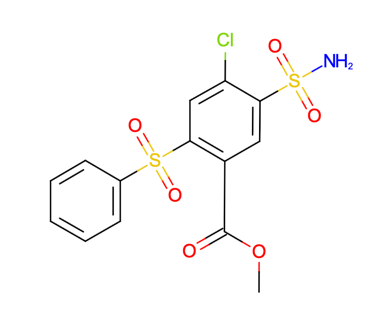 | 1.33 |
| *h*CA II | 6SBL | 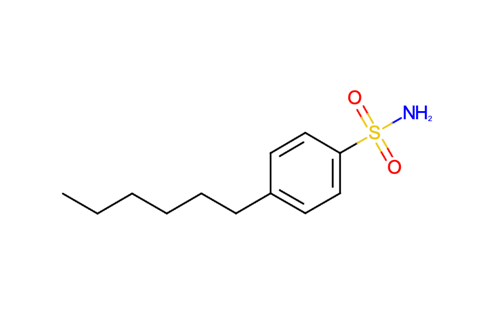 | 0.89 |
| *h*CA VII | 6SDT | 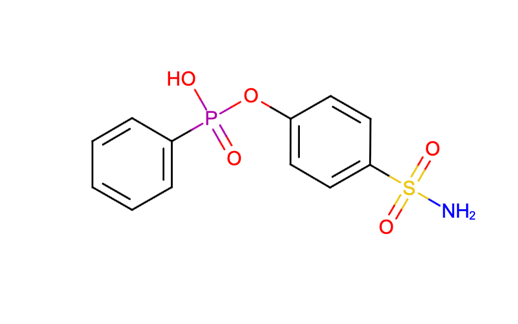 | 1.71 |
| *h*CA IX | 5FL4 | 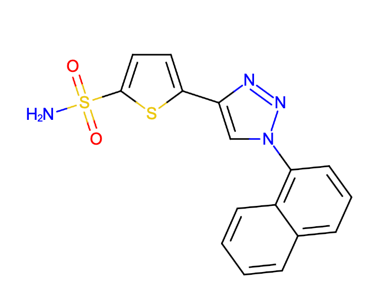 | 0.82 |
| *h*CA XII | 5MSA | 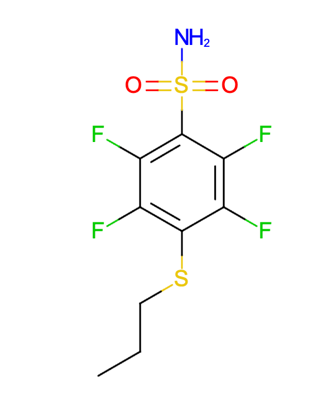 | 1.23 |

**Table S2**. G-score and ΔG_bind_ values of Acetazolamide (AAZ) complexed to all *h*CA isoforms used in this study. G-score and ΔG_bind_ values are reported as Kcal/mol.

| ***h*CA**  **isoforms** | **G-score**  **(*Kcal/mol*)** | **ΔG_bind_**  **(*Kcal/mol*)** |
| --- | --- | --- |
| *h*CA I | -7.20 | -42.03 |
| *h*CA II | -7.54 | -38.13 |
| *h*CA VA | -4.48 | -25.22 |
| *h*CA VII | -7.53 | -37.55 |
| *h*CA IX | -7.94 | -39.38 |
| *h*CA XII | -8.27 | -44.60 |

**Table S3**. Energetic components related to ΔG_bind_ (ΔG_Coul_, ΔG_lipo_, ΔG_solvGB_, ΔG_vdW_) for Acetazolamide (AAZ), silychristin and isosylibin A complexed with *h*CA VA and VII. All ΔG_bind_ values are reported as Kcal/mol.

|  | ***h*CA VA** | | | ***h*CA VII** | | |
| --- | --- | --- | --- | --- | --- | --- |
|  | **Acetazolamide (AAZ)** | **Silychristin** | **Isosylibin A** | **Acetazolamide**  **(AAZ)** | **Silychristin** | **Isosylibin A** |
| **ΔG_lipo_**  *(Kcal/mol)* | -1.33 | -14.82 | -14.79 | -2.49 | -16.64 | -18.83 |
| **ΔG_vdW_**  *(Kcal/mol)* | -16.89 | -38.08 | -39.72 | -22.94 | -40.21 | -39.50 |
| **ΔG_Coul_**  *(Kcal/mol)* | -50.84 | -15.44 | -32.32 | -77.97 | -52.50 | -33.71 |
| **ΔG_SolvGB_**  *(Kcal/mol)* | 44.53 | 41.70 | 50.00 | 64.83 | 60.32 | 52.87 |


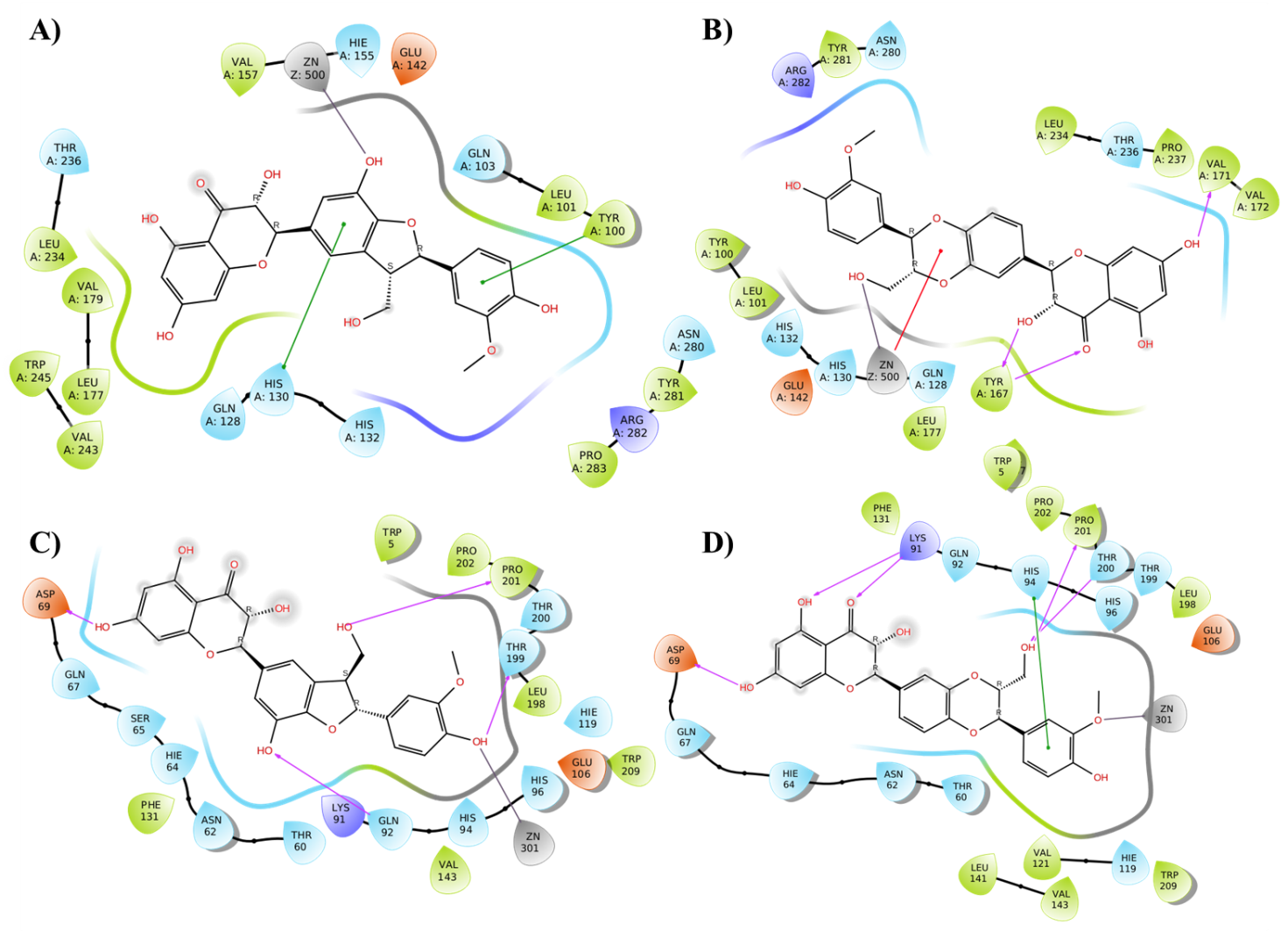


**Figure S1**. 2D representation of *h*CA VA and *h*CA VII complexed with **(A, C)** silychristin and **(B, D)** isosilybin A, respectively. H-bonds, electrostatic, and stacking interactions are illustrated as magenta, red, and green lines, respectively.


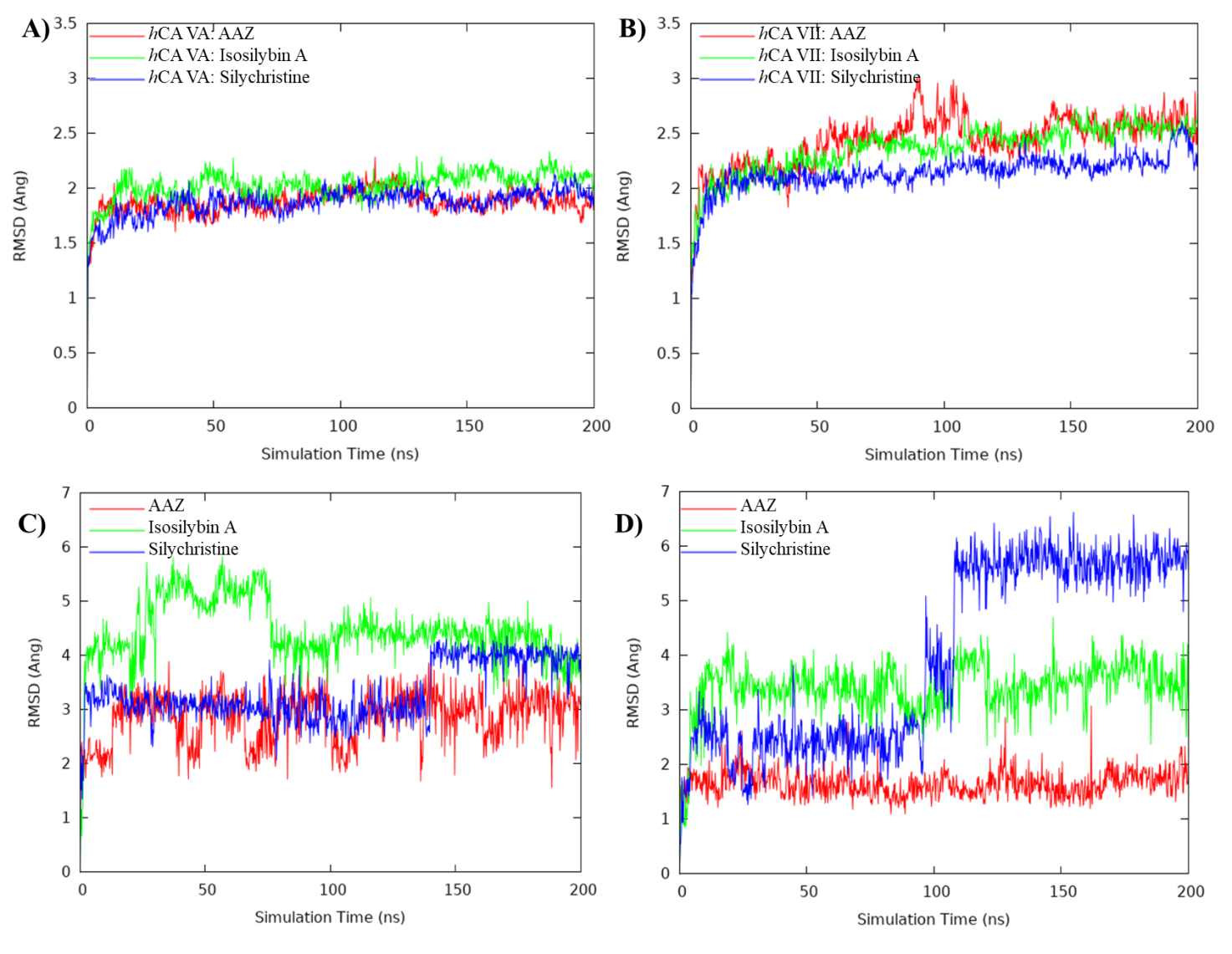


**Figure S2**. **A-B)** Plot of the RMSD values calculated on the heavy atoms of *h*CA VA (A) and *h*CA VII (B) over 200 ns of MD simulations. The complexes studied include AAZ (red line), isosilybin A (green line), and silychristine (blue line). **C-D)** Plot of the RMSD values calculated on the heavy atoms for AAZ (red line), isosilybin A (green line), and silychristine (blue line) complexed with *h*CA VA (A) and *h*CA VII (B) over 200 ns of MD simulations.
